# Foreign Body Endoscopy Experience of a University Based Hospital

**DOI:** 10.4021/gr517w

**Published:** 2013-03-09

**Authors:** Eiad Nassar, Rabi Yacoub, Dany Raad, Jason Hallman, Jan Novak

**Affiliations:** a Department of Gastroenterology, State University of New York at Buffalo, NY, USA; b Department of Medicine, State University of New York at Buffalo, NY, USA

**Keywords:** Endoscopy, Foreign bodies, Esophagus

## Abstract

**Background:**

Guidelines support endoscopic removal of certain gastric FB and all FB lodged in the esophagus. We aim to report our experience on endoscopic foreign bodies (FB) removal in order to aid in the formation of future guidelines regarding this subject.

**Methods:**

Retrospective analysis of one hundred forty-four cases of FB removal involving 43 patients who underwent esophagogastroduodenoscopy (EGD) for FB removal from January 2005 through December 2010 in a university-based hospital. To evaluate to outcome of endoscopic FB removal, cost of procedures and complications.

**Results:**

Of all FB removal cases, 23 (53%) were males, with total mean age of 26.4 ± 11.3 years. Only 20% were performed on an outpatient bases. Abdominal x-ray was obtained to confirm ingestion of FB in 83%, and computed tomography scan was performed in 13%. Most procedures were performed in operation room (59%) while only 21% of the cases were performed in endoscopy lab. General anesthesia was used in 58%, while monitored anesthesia care in 28%. Average time to EGD was 17.14 hours. No major complications due to procedure were reported. Minor trauma and erosions due to FB were reported in 14%. FB extraction was unsuccessful in only three cases, and one case required surgical intervention. Cost of all procedures was over 430, 000 dollars with mean of 2,990 dollars for procedure.

**Conclusion:**

Endoscopic retrieval is effective and safe procedure, but utilizes significant hospital resources.

## Introduction

Foreign body ingestion (FBI) is a common problem in the United States with an incidence of 120 per 1 million population, and accounting for 1500 annual fatalities [1]. The majority of FBI cases occur in the pediatric population mostly in children between 6 months and 6 years [2-4]. The occurrence of FBI in adults is almost strictly limited to patients with known psychiatric disorders or mental retardation [5], and prisoners seeking secondary gain by access to a medical facility [2, 6, 7].

Although most of the foreign bodies (FB) that reach the gastrointestinal (GI) tract will pass spontaneously [8], 10% to 20% will require non-operative intervention, and 1% or less may require surgery [2, 9, 10]. Management is influenced by several factors including the patient’s age and clinical condition, the type of foreign body as well as the anatomic location in which the object is lodged and the technical abilities of the endoscopist [11, 12]. Conservative outpatient management is indicated in almost all instances in which small, blunt objects have entered the stomach, with most objects passing within 4 to 6 days [3, 4]. Sharp pointed objects carry a risk of complication as high as 35% when lodged in the stomach [13], and therefore should be retrieved endoscopically when it can be accomplished safely [2]. Emergent endoscopic removal is recommended for disc batteries and shaped objects lodged in the esophagus [14].

The first report of foreign body removal using a flexible endoscope was published in 1972 [15], and since then there has been an increasing application of this method. Prior studies have shown that the equipment that should be readily available includes rat tooth and alligator forceps, polypectomy snare, polyp grasper, retrieval net, overtubes of esophageal and gastric lengths, and a foreign body protector hood [16]. The success rate of endoscopic removal of foreign bodies has been reported between 90% and 98.8%, with failures reported mostly in cases of dental prostheses, or complex and ultralong objects [2, 9, 17]. The highest complication rate reported for endoscopic treatment is 5%, with complications including mucosal laceration, bleeding, pyrexia, and esophageal perforation [17-19]. In addition to the complication rate, FBI also carries a large burden in terms of cost on patients, hospitals and third party payers. A recent study by Huang et al evaluated 305 FB removals in 33 patients and reported a total cost of over 2 million dollars over a span of 8 years spent in the management of FBIs [20].

The purpose of our study is to report our experience in a tertiary medical center on endoscopic foreign body removal in order to aid in the formation of future guidelines regarding this subject.

## Materials and Methods

We retrospectively identified all patients who underwent endoscopy for foreign body removal between January 2005 and July 2011 at Erie County Medical Center, through review of medical records and endoscopy reports from the gastroenterology department. Patients with incomplete or missing records were excluded. Data were collected on patients’ demographics, date of procedure, duration between image diagnosis and performing therapeutic esophagogastroduodenoscopy (EGD), location of procedure (emergency room, endoscopy suite or operating room), and duration of procedure. The types of FB ingested, tools used during endoscopy including the use of overtube for esophageal protection, type of sedation, use of endotracheal intubation for airway protection, complication from the FB ingestion or the procedure, underlying psychiatric disease and an estimate of procedure cost were also analyzed. This study was approved by the University at Buffalo Health Sciences Institutional Review Board.

## Results

### Patient demographics

**Figure 1:**
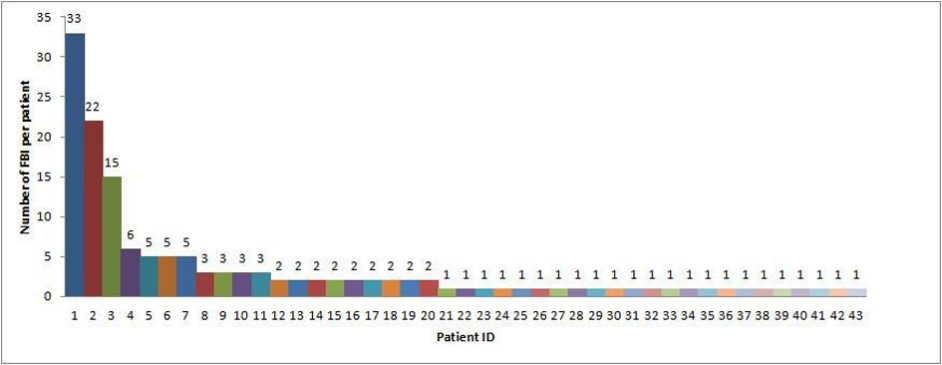
CT scan showing an incarcerated right femoral hernia.

A total of 144 endoscopic FB removal was performed on 43 patients, of whom 23 (53%) were males and 20 (47%) were females (Table 1). The mean age at the time of ingestion was 26.4 years (range 14 - 71 years). Figure 1 shows the number of individual patients responsible for the 144 cases. Overall there was an average of 3.3 cases per patient. One patient was accounted for 33 cases (23%). Twelve patients (28%) were admitted from the prison. The majority of patients (79%) had psychiatric diseases, including psychosis (28%), depression/bipolar (32%), PTSD (5%), and dementia (14%).

**Table 1:** Patient Demographics

	Number of patients (%)
Males/females	23 (53)/20 (47)
Age Group	
0 - 18	10 (23.2)
19- 25	7 (16.3)
26 - 45	17 (39.5)
> 45	9 (21)
Prisoners	12 (28)
Psychiatric Diseases	34 (79)
Psychosis	12 (28)
Depression/Bipolar	14 (32)
PTSD	2 (5)
Dementia	6 (14)

#### Diagnosis and management

The diagnosis of FBI was confirmed in 83% of the cases with a plain abdominal x-ray; a computed tomography (CT) scan was needed in only 13% of the cases. The diagnosis was made with no imaging studies after a witnessed incident in 4% of the cases. EGD was done on an inpatient basis in 80% of the cases with the remainder of the procedures done as an outpatient. The average time to endoscopy was 17.14 hours with a range between 45 minutes up to 10 days, as two patients initially refused the procedure.

#### Types of foreign bodies and endoscopic removal

The most commonly ingested foreign bodies were pens (24 cases), pencil (18), plastic utensils (12), razor blades (10), toothbrushes (9), screws (8), and markers (8) (Table 2). Other objects included, but were not limited to an albuterol can, sewing needles, screw driver, scissors, light bulb, incentive spirometer, and a lighter. The most commonly used endoscopic tools for extraction of the foreign body were snare (81 cases), rat tooth forceps (49), and roth net (13). Overtube was used in 43 sharp FBI cases to protect the esophagus while the hood was used in 18 cases. A 2 channel scope was used to remove long objects in 8 cases (Table 3).

**Table 2:** Types of Foreign Bodies Swallowed

Pen	24
Pencil	18
Plastic Utensil	12
Razor blade	10
Toothbrush	9
Screw	8
Marker	8
Paper clip	6
Batteries	5
Zipper	4
Thermometer cover	4
Nail	4
Hair pins	4
Wrist band	3
Tooth paste tubing	3
Hair clips	3
Coin	3
Others*	48

**Table 3:** Characteristics of Endoscopy

	Number of patients (%)
In-patient admissions	115 (80)
Time to EGD (hours)	
0 - 3	24 (18)
3 - 6	41 (30)
6 - 12	10 (7)
12 - 24	36 (26)
24 - 48	19 (14)
> 48	7 (5)
Site of EGD	
Operating Room	85 (59)
Endoscopy Suite	30 (21)
Emergency Room	29 (20)
Type of anesthesia	
General Anesthesia	83 (58)
Moderate Anesthesia with deep sedation	40 (28)
Moderation Sedation	21 (14)
Intubation	104 (72)
Endoscopic tools used for removal	
Snare	81
Rat tooth forceps	49
Roth retrieval net	13
Accessory endoscopy tools	
Overtube	43
Hood	18
2 channel scope	8

#### Location of procedure and type of sedation used

Endoscopic procedure for foreign body removal was performed most commonly in the operating room (85 cases, 59%). Only 21% of the cases (n = 30) were performed in the endoscopy suite and 20% (n = 29) were performed in the emergency room. The standard sedation used for endoscopic procedures in our institution is moderate sedation and is usually administered by the nurse, however the majority of FB removal cases were performed under general anesthesia (83 cases, 58%), and monitored anesthesia with deep sedation was used in 40 cases (28%). Overall, 104 cases (72%) required intubation during the procedure.

#### Outcomes and complications

Complications of the ingestion per se were observed in 20 cases (14%) including erosion (14) and ulceration (6). Foreign body extraction was unsuccessful in only three cases, and one case required surgical intervention. Complications from the procedure include erosion and laceration which occurred in 3.5% of the cases.

### Cost analysis

**Figure 2:**
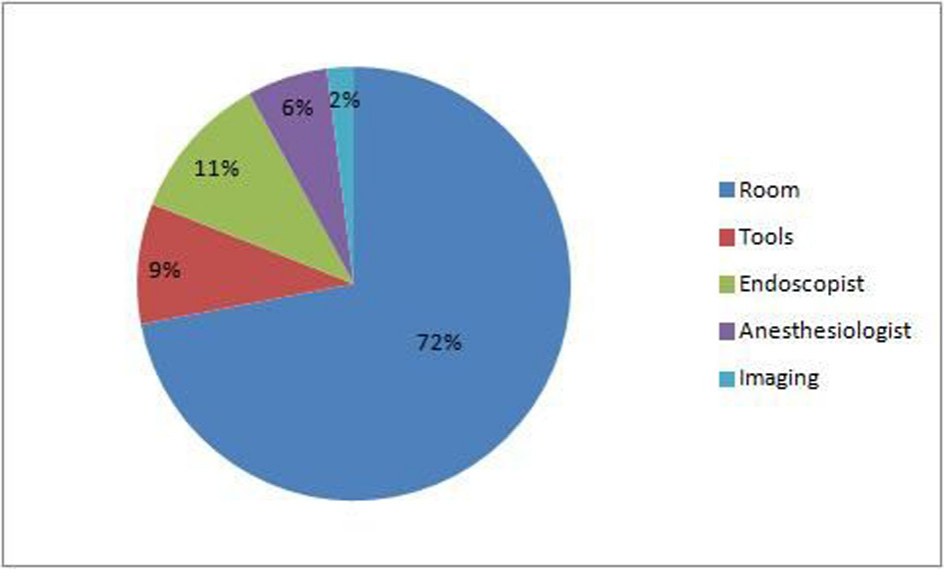
Break-down of total cost of endoscopic procedures for FBI over 5 years.

The total cost of all 144 endoscopic procedures performed for FBI over the 5 years was $434.322. Figure 2 shows the break-down of the total cost. The average cost of the procedure was $2,990 (range $1,175 - $4,291). Medicare reimbursement for the endoscopist is $341 and $219 for the anesthesiologist. The reimbursement based on the site is 727$ for the emergency room, 3,200$ for the operation room and $676.75 for the GI suite. The average cost of the endoscopy tools is $257. Since our institution is a county medical center, most of the patients are covered by medicare and the mean re-imbursement was $603 per procedure.

## Discussion

Intentional FBI is a frequent problem encountered in our institute which is associated with a significant cost and use of resources. The majority of the literature has focused on accidental FBI’s especially among children and young adults, with only few studies reporting intentional FBI’s. A recent study by Patta et al examined 262 cases of FBI’s in Los Angeles of which 92% were intentional, 85% involved psychiatric patients, and 84% occurred in patients with prior ingestions [5]. Another study identified 305 cases of FBI’s in 33 patients, of which 79% carried psychiatric diagnoses [20]. In our study, 79% of the cases involved psychiatric patients and 28% were prisoners. All but one case was intentional. The reasons and motivations for FBI vary; with an element of impulse disorder, affective dysregulation and suicidal behavior as attributing factors among patients with psychiatric disorders. FBI is also considered a form of self-injurious behavior with motivations such as acting out, internal distress communication and secondary gain [21].

Clinical history and examination are usually enough to establish the diagnosis in witnessed and accidental FBI’s; biplane radiographs can identify most true foreign objects with the exception of wood, plastic, glass, thin metal objects and fish or chicken bones [14]. The diagnosis of FBI in our study was made using plain radiographs in the majority of the cases. Handheld metal detectors have been reported in the literature as screening tools for FBI’s in the pediatric population [22]. CT scan may be useful in some cases but a contrast examination should not be performed routinely to avoid aspiration and compromise of subsequent endoscopy by coating the foreign object and the esophageal mucosa [12].

The current American Society of Gastrointestinal Endoscopy (ASGE) guidelines state that urgent endoscopic intervention is indicated in FBI’s for a sharp object, a disc battery, or any object that causes high-grade obstruction in the esophagus; while objects without high-grade obstruction or acute distress could be left in the esophagus but no longer than 24 hours [14]. Although many institutions apply endoscopic removal as a standard practice, the guidelines recommend conservative management for small blunt objects in the stomach. Exceptions include sharp objects due to the high risk of complications, and long objects (6 - 10 cm) as they are unlikely to pass spontaneously. The average time to endoscopy in our study was 17.14 hours, and it was related to the wide range between 45 minutes and 10 days, with the majority of the procedures performed within 24 hours. This is similar to published literature [5].

The preferred modality for removal of foreign bodies remains endoscopic intervention with success rates reported between 83% up to 99% [2, 5, 10, 13]. In our study, endoscopy was successful in 97.92% of the cases. Polypectomy snare, rat tooth forceps, and roth net were all tools used successfully to remove foreign objects in our center. Rubber hood, gastric and esophageal overtubes were used occasionally for esophageal protection; however, the choice of any of these tools should be used based on the foreign body and the operator experience [14]. Foley catheter use under fluoroscopy has also been used to extract foreign bodies in the past. One study reported successful removal in more than 2,500 patients using foley catheters, with only a 0.4% complication rate [23]. However, this technique does not allow inspection of the esophageal mucosa and therefore was not used in our study.

Endoscopic removal is considered a very safe procedure with morbidity rates less than 1% [2]. The most serious complication is perforation with a published perforation rate of 0.34% [24]. In our study, the procedure related complication rate was 2.08%, and was due to long and sharp FB’s. All complications were treated conservatively, and none of the cases necessitated surgery. No serious morbidity or mortality was found, similar to what has been documented in previous studies [2, 13, 18].

The use of general anesthesia with endotracheal intubation is required in endoscopic removal of foreign bodies when working with sharp and pointed objects as well as batteries [2]. In our practice the overuse of general anesthesia with endotracheal intubation (72% of cases) was due to the anesthesiologist’s preference and in consensus with the endosopist based on patients’ cooperation and the nature on the ingested foreign body.

The average cost of endoscopic removal of FB was estimated in our study to be $2,990 per case, which adds up to more than $400,000 for hospital costs over the 5 years representing only the cost of procedure. This huge burden and financial implication has been addressed by several studies in the past. Huang et al proposed an algorithm in an effort to limit the costs of management of these cases that entailed triage of patients based on the facility they are admitted from, types of foreign body and anatomic location, as well as the complications from the ingestion [20].

The majority of the literature has focused on accidental FBI, especially in children. This is one of the few studies that reports intentional FBIs in adults, and highlights the cost incurred in the management of these patients. However, our study has many limitations. First, the study is a retrospective chart review, and lacks important information on follow-up and long-term complications. Second, the patient population includes prisoners, where ingestion of foreign bodies has the secondary gain of hospital admission, causing the misleading of physicians by giving falsified medical information making it difficult to differentiate accidental from intentional ingestion.
